# Cerebral Blood Flow Alterations in High Myopia: An Arterial Spin Labeling Study

**DOI:** 10.1155/2020/6090262

**Published:** 2020-01-09

**Authors:** Huihui Wang, Shanshan Li, Xi Chen, Yanling Wang, Jing Li, Zhenchang Wang

**Affiliations:** ^1^Department of Radiology, Beijing Friendship Hospital, Capital Medical University, China; ^2^Department of Ophthalmology, Beijing Friendship Hospital, Capital Medical University, China

## Abstract

**Objective:**

The aim of this study was to explore cerebral blood flow (CBF) alterations in subjects with high myopia (HM) using three-dimensional pseudocontinuous arterial spin labeling (3D-pcASL).

**Methods:**

A total of sixteen patients with bilateral HM and sixteen age- and sex-matched healthy controls (HCs) were recruited. All subjects were right-handed. Image data preprocessing was performed using SPM8 and the DPABI toolbox. Clinical parameters were acquired in the HM group. Two-sample *t*-tests and Pearson correlation analysis were applied in this study.

**Results:**

Compared to HCs, patients with HM exhibited significantly increased CBF in the bilateral cerebellum, and no decreases in CBF were detected in the brain. However, no relationship was found between the mean CBF values in the different brain areas and the disease duration (*P* > 0.05).

**Conclusions:**

Using ASL analysis, we detected aberrant blood perfusion in the cerebellum in HM patients, contributing to a better understanding of brain abnormalities and brain plasticity through a different perspective.

## 1. Introduction

High myopia (HM) is a serious public health issue; this condition is increasingly prevalent among 2.9% of the global population and has affected 10-20% of young adults in East and Southeast Asia [1, 2]. High myopia is defined as having an ocular refractive diopter lower than -6.00 diopters (D) or an axial length larger than 26 mm and is characterized by blurred vision. Visual abnormalities could result in aberrant neural activity and neural plasticity, which have been demonstrated in some studies [3, 4]. High myopia (HM) is also termed “pathological myopia” or “degenerative myopia” and shows a widespread trend toward the development of pathological and degenerative changes in the neurosensory retina, retinal pigment epithelium (RPE), sclera, and choroid and notably, progression to visual impairment [5, 6]. This condition also has an elevated rate of ocular complications in the macula, peripheral retina, and optic nerve, and an increase in intraocular pressure (IOP) often accompanies it [7, 8]. Additionally, HM is an independent risk factor for glaucoma and is frequently associated with atypical retinal nerve fiber layer (RNFL) defects and nerve vulnerability [9, 10]. Glaucoma, characterized by retinal impairment, has been found to be accompanied by cortical metabolism alterations [11], abnormal neuronal activity [12], and brain tissue loss [4, 13, 14]. Nevertheless, the degeneration of the lateral geniculate nucleus (LGN) and primary visual cortex has been found in patients with optic nerve damage and increased IOP [15, 16]. These findings support the fact that the fundus structure, especially the retina and optic nerve, is closely related to the brain, especially vision-related areas. Some recent studies mainly focused on changes in the orbital cavity [5, 17–19], but the pathophysiology and progression of high myopia are still unclear. It is also unknown whether the complicated physiological changes in the brain are involved in the occurrence and development of the disease. Due to the long history of hypopsia and high incidence of fundus lesions in HM, it is necessary and meaningful to explore metabolism and functional abnormalities in the brain in HM.

Arterial spin labeling (ASL) perfusion MRI is a relatively new and noninvasive imaging modality that uses magnetically labeled water in arterial blood as an endogenous tracer to estimate brain perfusion at the tissue level [20]. Due to its higher labeling efficiency and intrasubject signal-to-noise ratio (SNR) compared to those of other ASL imaging techniques [21–24], the pseudocontinuous ASL (pcASL) technique is currently recommended in clinical studies and has become an increasingly popular tool among researchers [21, 25, 26]. ASL can visualize and quantify cerebral blood flow (CBF), and CBF could be a credible physiological marker for metabolism and neuronal activity [27]. Perfusion and metabolism in the brain are closely coupled [28, 29]. A number of studies have demonstrated that there is a strong correlation between brain perfusion measured on ASL images [30–32] and brain metabolism quantified with fluorine 18 fluorodeoxyglucose (FDG) positron emission tomography (PET) [33–35]. Compared to the traditional metabolic and perfusion techniques such as PET, dynamic susceptibility contrast (DSC), and dynamic contrast-enhanced (DCE) MR imaging, which are highly invasive, less widely available, and more expensive [36, 37], ASL is noninvasive, relatively straightforward to implement without the use of a contrast agent or radioisotopes [38]. In addition, blood is a critical element for the normal physiological activity of nerve cells [39]. Therefore, ASL can be a credible biomarker to reflect neuronal activity, which has been widely used in research on Alzheimer's disease [40, 41], epilepsy [42, 43], and depressive disorders [44]. With regard to the technology reflecting brain activity, blood oxygen level-dependent (BOLD) fMRI is becoming progressively more popular. However, the BOLD signal is an indirect measure of the vascular response rather than an absolute measure of neuronal activation and is susceptible to vascular factors [36]. ASL is less sensitive to the influence of confounding factors and reveals the level of parenchymal perfusion, which can be a more accurate measurement of neuronal function [45]. Its practical advantages make this technique an exceptionally powerful tool for exploring subtle changes in the brain for the purpose of diagnosis and treatment assessment in clinical research studies [46, 47].

The quantitative perfusion index holds promise as an objective biomarker for tracking illness progression and the effects of therapy in a variety of developmental disorders [20, 46]. Consequently, we aimed to apply a three-dimensional pseudocontinuous arterial spin labeling (3D-pcASL) sequence to explore CBF alterations in the brain in high myopic subjects, hoping to gain key insights into the pathophysiological mechanism of the disease from a novel perspective.

## 2. Materials and Methods

### 2.1. Subjects

A total of sixteen right-handed patients with bilateral HM (7 males and 9 females) from Beijing Friendship Hospital, Capital Medical University, were enrolled in the study, and ages ranged from 25 to 65 years old. HM was defined as refractive diopter less than -6.00 D or the axial length larger than 26 mm. The exclusion criteria were as follows: any other ocular diseases (e.g., amblyopia, strabismus, glaucoma, and optic neuritis); unilateral HM; psychiatric disorders or cerebral infarction diseases; and any systemic diseases that may influence the results, such as hypertension and diabetes. All HM patients had vision corrected with glasses and had at least one complication due to high myopia, such as retinal atrophy degeneration, retinal detachment, or a macular hole.

Sixteen right-handed healthy controls (HCs) (7 males and 9 females) with uncorrected visual acuity (VA) > 1.0 were also recruited. The age of the HCs ranged from 28 to 65 years old. The exclusion criteria were as follows: any ocular disease; vascular disease; psychiatric disorders (e.g., depression, schizophrenia); any systemic diseases that may influence the brain blood perfusion, such as hypertension and diabetes; and MRI ineligibility (e.g., cardiac pacemaker, replacement heart valves, or implanted metal devices).

This study was approved by the medical research ethics committee of Beijing Friendship Hospital, Capital Medical University. Written informed consent was obtained from all subjects prior to enrollment.

### 2.2. MRI Data Acquisition

MRI data were acquired using a GE MR750 Discovery 3 T MRI scanner (General Electric, Milwaukee, WI, USA) equipped with an 8-channel head coil. During the examinations, we offered foam pads and earplugs to reduce noise and head motion, and all subjects were instructed to stay relaxed and motionless with the eyes closed.

The 3D-pcASL sequence was performed using a 3D gradient and fast spin-echo stack of spiral arterial labeling. Each ASL scan consisted of 36 pairs of labeled and control slices, which were obtained with the following sequence parameters: slice thickness = 4.0 mm, FOV = 240 mm × 240 mm, reconstruction matrix = 512 × 8 (K space filling technique), TE = 10.7 ms, TR = 5337 ms, postlabeling delay (PLD) = 2525 ms, spiral arms = 8, points per arm = 512, and number of excitations (NEX) = 3. The total acquisition time for the ASL scan was approximately 5 minutes.

### 2.3. Image Processing

**Figure 1:**
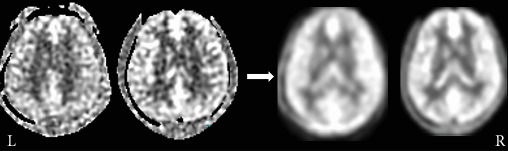
Original and smoothed CBF maps from control and HM groups, respectively.

All images were preprocessed with statistical parametric mapping (SPM8, http://www.fil.ion.ucl.ac.uk/spm) and the data processing and analysis of brain imaging (DPABI, http://rfmri.org/dpabi) toolbox implemented in MATLAB (Version R2013a; MathWorks, Natick, MA, United States). The CBF maps were converted to ASL MRI images to identify changes in regional perfusion, and the specific calculation procedures have been described previously [48]. Image preprocessing was conducted as follows: (1) normalization: a one-step registration method was applied, and all subjects' CBF maps were coregistered to a PET-perfusion template in the Montreal Neurological Institute (MNI) space and resampled with a 2 mm × 2 mm × 2 mm voxel size using the SPM toolbox; (2) standardization: the normalized CBF maps were standardized with the mean division method using the DPABI package; and (3) smoothing: standardized CBF images were then smoothed with an 8 mm Gaussian kernel to reduce interindividual differences and increase the SNR (Figure 1).

### 2.4. Statistical Analysis

The differences in demographic and clinical variables between the HM and HC groups were analyzed using SPSS 22.0 software (SPSS, IBM Corporation, Armonk, NY, USA). Independent sample *t*-tests and Fisher's exact test were applied with the statistical threshold set at 0.05.

The voxel-wise difference in the smoothed CBF between two groups was compared with a two-sample *t*-test using the SPM8 toolbox. During statistical analysis, an explicit cerebrum and cerebellum mask was added to eliminate the effect of scalp tissue from each smoothed CBF image and to prevent the increased CBF from the cerebellum to “leak” into the cerebrum, which could avoid the possibility to form an inaccurate larger significant cluster. Multiple comparisons were corrected using a family-wise error (FWE) method with a threshold of 0.001. The results were considered to be statistically significant when *P* < 0.05.

The results were visualized by the xjView toolbox (xjView9.5, http://www.alivelearn.net/xjview/) and BrainNet Viewer (BrainNet Viewer 1.61, http://www.nitrc.org/projects/bnv/). Each cluster with significant differences was saved and used as a mask for the purpose of the subsequent region of interest- (ROI-) based analyses. Then, the CBF values of the ROIs in the HM subjects were extracted with the xjView toolbox to analyze the correlation between the CBF value and clinical parameters.

## 3. Results

### 3.1. Demographic and Clinical Characteristics of the Subjects

The baseline data of thirty-two subjects (16 with high myopia and 16 with normal controls) are shown in Table 1. There were no significant differences in age or gender between the HM and HC groups (*P* = 0.818, *P* = 1).

### 3.2. CBF Changes in HM Patients

**Figure 2:**
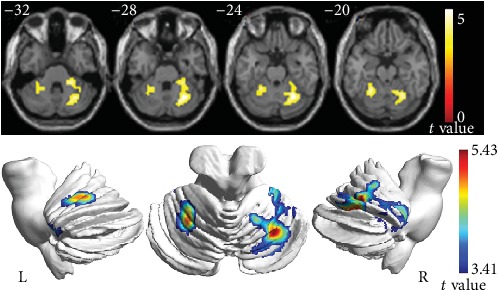
Blood perfusion of the brain in the HM and HC groups. Voxel-based analysis indicates the brain regions with significant group differences in the normalized CBF; the brain regions shown in the figure represent regions with increased CBF (*P* < 0.05, FWE corrected, cluster size > 584). Abbreviations: CBF: cerebral blood flow; HCs: healthy controls; L: left; R: right; HM: high myopia; FEW: family-wise error.

In the voxel-based analysis, the CBF differences between the HM patients and the matched healthy subjects are shown in Figure 2 and Table 2. Compared with HCs, HM patients exhibited increased CBF in the bilateral cerebellum (all *P* < 0.05, FWE corrected), but no regions showed significantly decreased CBF.

### 3.3. Correlation between the CBF Value and Clinical Parameters

The CBF values of each cluster with significant differences were extracted (Table 3), and Pearson's correlation analysis was performed to assess the association between the course of the disease and CBF values in HM patients (*P* < 0.05 was considered statistically significant). However, there was no significant correlation between the CBF values of the bilateral cerebellum in HM subjects with the duration of the illness (R: *P* = 0.092, L: *P* = 0.264).

## 4. Discussion

Studies of CBF in ophthalmic diseases are limited [11, 49]. To our knowledge, this is the first study to analyze the regional brain perfusion alterations of HM patients using the 3D-pcASL technique. To our surprise, we did not find any alterations in the cerebrum, but we found increased CBF in the bilateral cerebellum of HM subjects compared to that of HCs.

Previous studies have demonstrated that blurred vision or vision loss causes aberrant morphological and functional changes in the cerebrum, especially in visual-related areas. Noppeney et al. [50] discovered that early blind individuals had decreased gray matter (GM) and white matter (WM) volumes in the visual system and increased WM volumes in the sensory-motor system. Mirzajani et al. [51] reported that severe blurring caused by lens-induced HM could cause a BOLD signal intensity decrease in the visual cortex. Guo et al. [52] observed that HM subjects showed a decreased amplitude of low-frequency fluctuation (ALFF) in the bilateral frontal lobe, right parietal lobe, and right middle temporal lobe in the eyes-closed condition compared to that in the emmetropia group. Zhai et al. [53] detected decreased functional connectivity between the ventral attention and frontoparietal control networks. Some studies of other ophthalmology diseases that involve fundus damage were also reported. Jiang et al. [54] found that POAG patients showed decreased GM density in the visual cortex. Wang et al. [55] showed that POAG individuals had reduced CBF in the visual cortices, which was relevant for the severity of POAG. There was also evidence indicating the relationship between the degree of visual field loss and the altered blood perfusion in the visual cortex or primary visual cortex in POAG patients [11]. However, there were no perfusion changes observed in our study, which demonstrated that HM subjects have no metabolic and functional alterations in the cerebrum. In addition, although patients in our study had fundus damage, we did not find any changes in visual-related areas that mimicked those observed in glaucoma patients. This discrepancy might be ascribed to the relatively thin cortex which would weaken the CBF effect in visual areas [56, 57]. The different criteria for HM patient grouping and technical methods may also account for the divergent results. Additionally, a larger sample size study was required for further investigation to acquire a more robust conclusion.

Increased CBF in the cerebellum of HM patients indicated that the cerebellum may play a vital role in vision formation and transmission, which had long been neglected. However, the mechanism remains unknown in HM patients. A possible explanation for this finding could be ascribed to the impaired function of ocular adjustment and reflex, which is partly controlled by the cerebellum, and the subsequent compensatory blood perfusion in HM patients. Ocular adjustment and reflex, which include saccade, smooth pursuit movement, fixation, accommodation, and the vestibuloocular reflex, are needed to maintain clear images on the retina [58]. However, HM patients usually present with reduced ocular adjustment and reflex abilities involving low adjustment sensitivity, adjustment lag, and decreased adjustment amplitude [59, 60]. Previous studies have shown that the cerebellum plays an important role in motor control and perception. Agarwal et al. [58] and Hayakawa et al. [61] suggested that the cerebellum contributes to the precision of eye movements involving saccadic eye movements, smooth pursuit movements, and related reflexes such as the vestibuloocular reflex and accommodation reflex. Interestingly, some studies found that posterior circulation arterial embolism could cause blindness [62], and cerebellar lesions are often combined with high myopia [63, 64], which may suggest that the cerebellum plays a vital role in the occurrence, development, and progression of high myopia. Function-related studies have further confirmed the relationship between the cerebellum and visual function. Huang et al. [65] found that the mean diffusivity values in the bilateral cerebellum posterior lobe were significantly decreased in comitant strabismus patients. Chen et al. [4] reported that the left cerebellum anterior lobe had a higher regional homogeneity (ReHo) value in patients with primary angle-closure glaucoma (PACG) than in HCs. Dai et al. [66] detected abnormal connections between the cerebellum and the primary visual cortex in primary open-angle glaucoma (POAG) patients. Hu et al. [67] reported increased neural activity in the right cerebellum posterior lobe. In line with a previous study, our results showed increased blood perfusion, representing higher metabolism and neural activity in the cerebellum in high myopic patients. Mon-Williams et al. [68] and George and Rosenfield [69] reported that the adaptation of the visual system to blurred defocus can trigger neuronal compensatory processes in the visual cortex, which could presumably increase visual cortex activity and blood perfusion. Hence, we speculate that increased CBF in our study is a compensatory change in HM development.

Some older participants were involved in this study, so the possible aging effects on the reported alterations should be considered. It is necessary to understand the evolution of CBF in the brain during normal aging. However, this pattern of CBF alteration is still controversial. Du et al. studied 44 healthy participants ranging from 4 to 78 years old and found that after the age of approximately 20 years old, gray matter perfusion remained rather constant until the age of approximately 80 years old [34]. Soni et al. studied one hundred and sixty normal volunteers of varying ages (6–72 years) and observed a significant negative correlation between age and the mean GM CBF values [37]. In our study, to control for possible aging effects, we added age as a covariate when analyzing voxel-based CBF differences, and we ensured that the groups were age-matched. Hence, we are confident that the present results are not merely due to age.

There are several limitations in the current study. First, the sample size was small, which should be expanded in future research. Second, the range of refractive diopters in HM patients was broad, and more rigorous criteria are needed for a precise conclusion. Third, the mechanism of abnormal CBF clusters was only based on our hypothesis, which was not supported by concrete data and needs further interpretation. Fourth, as the present study is cross-sectional, we cannot completely address these perfusion patterns as a product of high myopia. Mild and moderate myopia subjects or future longitudinal studies in patients in different stages of the disease might be needed to prove the change law of cerebral blood flow.

## 5. Conclusion

In summary, high myopia patients exhibit increased CBF in the bilateral cerebellum, which may contribute to the understanding of HM-related brain metabolism, activity, and plasticity. However, the causality between high myopia and abnormal brain alterations still requires further investigation.

## Figures and Tables

**Table 1:** Demographic and clinical measurements of the subjects.

	HM (*n* = 16)	HCs (*n* = 16)	*P* value
Age	46.7 ± 13.6	47.7 ± 10.6	0.818^b^
Gender (male/female)	7/9	7/9	1^a^
Handedness	16 right-handed	16 right-handed	1^b^
HM duration (years)	32.6 ± 13.6	NA	NA
Refractive diopter_R (D)	−8.4 ± 5.5	NA	NA
Refractive diopter_L (D)	−8.4 ± 5.6	NA	NA
Axial length_R	28.0 ± 1.7	NA	NA
Axial length_L	27.9 ± 2.2	NA	NA

Data are presented as the range of min–max (mean ± standard deviation). Abbreviations: HM: high myopia; HCs: healthy controls; NA: not applicable; D: diopter; R: right; L: left. ^a^Fisher's exact test. ^b^Two-sample *t*-test.

**Table 2:** Brain regions with increased CBF in HM subjects compared to HCs.

Regions	Cluster size (voxels)	Peak *t* values	MNI coordinates		
			*x*	*y*	*z*
Cerebellum_R	1309	5.43	28	-62	-22
Cerebellum_L	584	5.21	-24	-54	-18

Abbreviations: CBF: cerebral blood flow; HM: high myopia; HCs: heathy controls; R: right; L: left; MNI: Montreal Neurological Institute.

**Table 3:** CBF values of significant clusters for each group.

Group	Mean CBF value (ml/100 g/min)	
	Cluster_R	Cluster_L
HM	54.2 ± 3.82	52.5 ± 3.72
HC	47.4 ± 3.38	45.5 ± 3.85

Abbreviations: CBF: cerebral blood flow; HM: high myopia; HC: heathy control; R: right; L: left.

## Data Availability

The ASL data and clinical information used to support the findings of this study are available from the corresponding authors upon request.
